# Supplementary material for: Identification of Lens culinaris defense genes responsive to the anthracnose pathogen Colletotrichum truncatum
Source: BMC Genet. 2013 Apr 30;14:31. doi: 10.1186/1471-2156-14-31 (PMC3666911; doi:10.1186/1471-2156-14-31)
Supplement: Additional file 1 — Defense associated lentil unigenes expressed at the in planta biotrophic-necrotrophic switch of Colletotrichum truncatum infection. [file 1471-2156-14-31-S1.docx]

| **EST ID** | **GeneBank Accession** | **Copy number** | **Length (nt)** | **Annotation (BLASTX)** | ***E*** **value** |
| --- | --- | --- | --- | --- | --- |
| ***Signal transduction*** **(159)** | | | | | |
| LT21-1561 | JG293480 | 1 | 1136 | ADP ribosylation factor 002 [*Elaeis guineensis*] | 4e-93 |
| LT21-1468 | JG293481 | 1 | 841 | ADP-ribosylation factor [*Arabidopsis lyrata*] | 3e-90 |
| LT21-1559 | JG293482 | 1 | 493 | ADP-ribosylation factor [*Arachis diogoi*] | 6e-39 |
| LT21-4750 | JG293483 | 1 | 922 | ADP-ribosylation factor-like protein 8A [*Zea mays*] | 3e-65 |
| LT21-3553 | JG293484 | 1 | 743 | ARF gtpase activator, putative [*Ricinus communis*] | 1e-43 |
| LT21-4406 | JG293485 | 1 | 645 | ARF gtpase activator, putative [*Ricinus communis*] | 8e-58 |
| Contig 345 | JG293486 | 2 | 1114 | Bzip transcription factor bzip110 [*Glycine max*] | 3e-19 |
| LT21-2796 | JG293487 | 1 | 1130 | Bzip transcription factor bzip114 [*Glycine max*] | 1e-47 |
| Contig 11 | JG293488 | 5 | 957 | Bzip transcription factor bzip41 [*Glycine max*] | 1e-26 |
| LT21-4706 | JG293489 | 1 | 507 | Bzip transcription factor bzip68 [*Glycine max*] | 1e-15 |
| LT21-3560 | JG293490 | 1 | 841 | Bzip transcriptional repressor ROM1 [*Phaseolus vulgaris*] | 6e-37 |
| LT21-2990 | JG293491 | 1 | 671 | MYB transcription factor MYB112 [*Glycine max*] | 9e-57 |
| LT21-830 | JG293492 | 1 | 977 | MYB transcription factor MYB37 [*Medicago truncatula*] | 1e-79 |
| LT21-4669 | JG293493 | 1 | 1175 | MYB transcription factor MYB92 [*Glycine max*] | 1e-51 |
| LT21-2113 | JG293494 | 1 | 1210 | MYB-like DNA-binding protein [*Catharanthus roseus*] | 2e-09 |
| LT21-1444 | JG293495 | 1 | 668 | Zinc finger DNA-binding protein [*Catharanthus roseus*] | 1e-05 |
| Contig 531 | JG293496 | 2 | 1222 | Zinc finger protein [*Carica papaya*] | 6e-68 |
| Contig 597 | JG293497 | 2 | 1042 | Zinc finger protein [*Cicer arietinum*] | 5e-59 |
| LT21-736 | JG293498 | 1 | 1145 | Zinc finger protein ZFP248 [*Arachis hypogaea*] | 3e-25 |
| Contig 23 | JG293499 | 2 | 711 | Zinc finger protein, putative [*Ricinus communis*] | 1e-34 |
| LT21-1282 | JG293500 | 1 | 713 | Zinc finger protein, putative [*Ricinus communis*] | 3e-10 |
| LT21-1876 | JG293501 | 1 | 480 | Zinc finger protein, putative [*Ricinus communis*] | 4e-27 |
| LT21-291 | JG293502 | 1 | 1042 | Zinc finger protein, putative [*Ricinus communis*] | 3e-52 |
| LT21-364 | JG293503 | 1 | 1104 | Zinc finger protein, putative [*Ricinus communis*] | 8e-37 |
| Contig 142 | JG293504 | 4 | 733 | Zinc finger protein, putative [*Ricinus communis*] | 4e-37 |
| LT21-979 | JG293505 | 1 | 1156 | Zinc finger protein-related [*Arabidopsis thaliana*] | 4e-26 |
| LT21-2365 | JG293506 | 1 | 1088 | Zinc finger, RING-type [*Medicago truncatula*] | 3e-51 |
| LT21-3829 | JG293507 | 1 | 1158 | Zinc finger, RING-type [*Medicago truncatula*] | 3e-82 |
| LT21-819 | JG293508 | 1 | 780 | Zinc finger, RING-type [*Medicago truncatula*] | 6e-47 |
| LT21-1829 | JG293509 | 1 | 735 | Zinc finger, RING-type; Thioredoxin-related [*Medicago truncatula*] | 1e-62 |
| LT21-996 | JG293510 | 1 | 1190 | Zinc finger, RING-type; Thioredoxin-related [*Medicago truncatula*] | 7e-61 |
| LT21-1527 | JG293511 | 1 | 1119 | Ethylene-responsive transciptional coactivator-like protein [*Retama raetam*] | 4e-30 |
| LT21-1687 | JG293512 | 1 | 765 | Ethylene-responsive transciptional coactivator-like protein [*Retama raetam*] | 2e-50 |
| LT21-3852 | JG293513 | 1 | 1161 | Ethylene-responsive transciptional coactivator-like protein [*Retama raetam*] | 1e-52 |
| Contig 193 | JG293514 | 2 | 1109 | Ethylene-responsive transciptional coactivator-like protein [*Retama raetam*] | 3e-42 |
| LT21-3265 | JG293515 | 1 | 649 | Putative Cys2-His2 zinc finger transcription factor [*Juglans regia*] | 2e-35 |
| LT21-2429 | JG293516 | 1 | 1170 | Transcription factor WRKY [*Lotus japonicus*] | 5e-45 |
| LT21-2400 | JG293517 | 1 | 1181 | WRKY transcription factor WRKY100630 [*Medicago truncatula*] | 1e-76 |
| Contig 585 | JG293518 | 2 | 1187 | Putative zinc-binding protein [*Platanus x acerifolia*] | 1e-77 |
| LT21-28 | JG293519 | 1 | 770 | Ring finger protein, putative [*Ricinus communis*] | 1e-63 |
| Contig 112 | JG293520 | 6 | 1127 | Translationally-controlled tumor protein, putative [*Ricinus communis*] | 3e-48 |
| Contig 355 | JG293521 | 2 | 1118 | Calcium binding protein [*Sesbania rostrata*] | 3e-57 |
| LT21-79 | JG293522 | 1 | 488 | Calcium binding protein [*Sesbania rostrata*] | 9e-32 |
| LT21-4645 | JG293523 | 1 | 717 | Putative calcium-binding protein [*Gossypium hirsutum*] | 2e-36 |
| LT21-4708 | JG293524 | 1 | 502 | Calcium-dependent protein kinase, putative [*Ricinus communis*] | 4e-59 |
| LT21-3452 | JG293525 | 1 | 698 | Calcineurin B [*Pisum sativum*] | 3e-59 |
| LT21-4556 | JG293526 | 1 | 961 | Calcineurin B-like-interacting protein kinase [*Pisum sativum*] | 3e-83 |
| Contig 617 | JG293527 | 2 | 1120 | Calmodulin [*Aquilaria microcarpa*] | 5e-78 |
| LT21-1910 | JG293528 | 1 | 1168 | Calmodulin [*Glycine max*] | 2e-20 |
| Contig 554 | JG293529 | 2 | 890 | Calmodulin 4 [*Daucus carota*] | 6e-76 |
| LT21-1006 | JG293530 | 1 | 1140 | Calmodulin 4 [*Daucus carota*] | 1e-78 |
| LT21-4526 | JG293531 | 1 | 1186 | Calmodulin binding protein, putative [*Ricinus communis*] | 1e-29 |
| LT21-4668 | JG293532 | 1 | 1181 | Calmodulin binding protein, putative [*Ricinus communis*] | 8e-60 |
| LT21-1506 | JG293533 | 1 | 578 | Calmodulin, putative [*Ricinus communis*] | 2e-52 |
| LT21-411 | JG293534 | 1 | 647 | Calmodulin-binding protein [*Arabidopsis thaliana*] | 1e-30 |
| LT21-1033 | JG293535 | 1 | 1168 | Calmodulin-binding protein, putative [*Oryza sativa*] | 1e-54 |
| Contig 533 | JG293536 | 2 | 907 | Calmodulin-like protein [*Pisum sativum*] | 3e-69 |
| Contig 55 | JG293537 | 2 | 793 | Putative calmodulin-like protein [*Populus x canadensis*] | 2e-46 |
| Contig 369 | JG293538 | 2 | 1193 | Gtpase [*Glycine max*] | 4e-85 |
| Contig 492 | JG293539 | 2 | 872 | GTP-binding protein [*Pisum sativum*] | 3e-83 |
| LT21-191 | JG293540 | 1 | 380 | GTP-binding protein typa [*Trifolium pratense*] | 1e-06 |
| LT21-4259 | JG293541 | 1 | 1181 | GTP binding protein [*Cichorium intybus x Cichorium endivia*] | 2e-95 |
| LT21-1558 | JG293542 | 1 | 1010 | Putative GTP-binding protein [*Cucumis sativus*] | 6e-79 |
| LT21-3746 | JG293543 | 1 | 1083 | Nucleolar GTP-binding protein, putative [*Ricinus communis*] | 1e-50 |
| LT21-3421 | JG293544 | 1 | 492 | ARA6; GTP binding / gtpase [*Arabidopsis thaliana*] | 3e-24 |
| LT21-1128 | JG293545 | 1 | 770 | Kinase family protein [*Arabidopsis lyrata* ] | 3e-51 |
| LT21-2945 | JG293546 | 1 | 339 | Kinase family protein [*Arabidopsis lyrata*] | 2e-13 |
| LT21-2091 | JG293547 | 1 | 1165 | Kinase, putative [*Ricinus communis*] | 5e-77 |
| Contig659 | JG293548 | 2 | 1152 | Kinase-related [*Arabidopsis thaliana*] | 5e-15 |
| LT21-2613 | JG293549 | 1 | 935 | MAP kinase phosphatase [*Datura metel*] | 2e-24 |
| LT21-4167 | JG293550 | 1 | 953 | MAP kinase 3 [*Pisum sativum*] | 3e-101 |
| LT21-2803 | JG293551 | 1 | 1187 | MAP kinase protein [*Cicer arietinum*] | 2e-39 |
| LT21-2590 | JG293552 | 1 | 743 | FUS5 (FUSCA 5); MAP kinase kinase [*Arabidopsis thaliana*] | 8e-10 |
| LT21-2022 | JG293553 | 1 | 1083 | MAPKKK5; Serine/threonine kinase [*Arabidopsis thaliana*] | 2e-29 |
| LT21-4400 | JG293554 | 1 | 768 | Mitogen-activated kinase kinase kinase alpha [*Lotus japonicus*] | 2e-46 |
| LT21-2077 | JG293555 | 1 | 1157 | Mitogen-activated protein kinase 2 [*Nicotiana tabacum*] | 3e-88 |
| Contig 99 | JG293556 | 2 | 829 | Putative mitogen-activated protein kinase 1 [*Medicago sativa*] | 6e-20 |
| LT21-321 | JG293557 | 1 | 489 | Patatin [*Medicago truncatula*] | 1e-23 |
| LT21-1661 | JG293558 | 1 | 902 | Patatin B2 precursor, putative [*Ricinus communis*] | 2e-53 |
| LT21-1159 | JG293559 | 1 | 1230 | Patatin-like protein 1 [*Nicotiana tabacum*] | 4e-62 |
| LT21-2805 | JG293560 | 1 | 464 | Phosphatase 2C family protein [*Arabidopsis lyrata*] | 3e-16 |
| Contig524 | JG293561 | 2 | 733 | Catalytic subunit of protein phosphatase 1 [*Vicia faba*] | 5e-20 |
| LT21-4642 | JG293562 | 1 | 1233 | Phospholipase C [*Arabidopsis lyrata*] | 2e-82 |
| LT21-1294 | JG293563 | 1 | 1169 | Phospholipase C, putative [*Ricinus communis*] | 4e-131 |
| Contig 30 | JG293564 | 2 | 1047 | Phospholipase like protein [*Arabidopsis thaliana*] | 3e-28 |
| LT21-2004 | JG293565 | 1 | 1200 | Protein farnesyltransferase beta subunit, putative [*Ricinus communis*] | 6e-99 |
| LT21-3126 | JG293566 | 1 | 1198 | Protein kinase [*Glycine max*] | 2e-88 |
| LT21-4494 | JG293567 | 1 | 911 | Protein kinase [*Glycine max*] | 2e-76 |
| LT21-4583 | JG293568 | 1 | 620 | Protein kinase [*Glycine max*] | 5e-06 |
| LT21-2072 | JG293569 | 1 | 845 | Protein kinase [*Medicago truncatula*] | 8e-49 |
| LT21-2215 | JG293570 | 1 | 658 | Protein kinase [*Medicago truncatula*] | 3e-35 |
| Contig 328 | JG293571 | 2 | 1175 | Protein kinase atmrk1, putative [*Ricinus communis*] | 7e-71 |
| LT21-1221 | JG293572 |  | 339 | Protein phosphatase 2C [*Medicago sativa*] | 4e-05 |
| LT21-2359 | JG293573 | 1 | 144 | Protein phosphatase 2C [*Medicago sativa*] | 2e-05 |
| LT21-3722 | JG293574 | 1 | 562 | Protein phosphatase 2C [*Medicago sativa*] | 2e-11 |
| LT21-4474 | JG293575 | 1 | 755 | Protein phosphatase 2C-like [*Medicago truncatula*] | 1e-47 |
| LT21-1688 | JG293576 | 1 | 1107 | Protein phosphatase type 2C [*Lotus japonicus*] | 3e-125 |
| Contig 511 | JG293577 | 3 | 1275 | Protein phosphatase-2C [*Mesembryanthemum crystallinum*] | 2e-61 |
| LT21-4479 | JG293578 | 1 | 419 | Protein-tyrosine phosphatase, mitochondrial precursor [*Ricinus communis*] | 2e-21 |
| LT21-864 | JG293579 | 1 | 1182 | Dual specificity protein phosphatase, putative [*Ricinus communis*] | 3e-53 |
| LT21-4181 | JG293580 | 1 | 1188 | Ras-related protein Rab-18 [*Zea mays*] | 4e-91 |
| LT21-1407 | JG293581 | 1 | 1173 | Ser/Thr protein kinase [*Lotus japonicus*] | 2e-46 |
| LT21-185 | JG293582 | 1 | 534 | Ser/Thr protein kinase [*Lotus japonicus*] | 7e-37 |
| LT21-3302 | JG293583 | 1 | 1208 | Ser/Thr protein kinase [*Lotus japonicus*] | 7e-50 |
| LT21-1764 | JG293584 | 1 | 1176 | Protein kinase - like protein [*Arabidopsis thaliana*] | 6e-71 |
| LT21-1711 | JG293585 | 1 | 1154 | Serine/threonine protein kinase, putative [*Ricinus communis*] | 1e-51 |
| LT21-3645 | JG293586 | 1 | 951 | Serine/threonine protein kinase, putative [*Ricinus communis*] | 2e-88 |
| LT21-2881 | JG293587 | 1 | 642 | Serine/threonine protein kinase-like protein [*Glycine max*] | 1e-36 |
| LT21-832 | JG293588 | 1 | 1168 | Serine/threonine-protein kinase cx32, putative [*Ricinus communis*] | 1e-98 |
| Contig580 | JG293589 | 2 | 1224 | Serine/threonine-protein kinase, putative [*Ricinus communis*] | 4e-68 |
| LT21-27 | JG293590 | 1 | 1162 | Serine-threonine kinase [*Persea americana*] | 3e-105 |
| LT21-1341 | JG293591 | 1 | 1229 | Serine-threonine protein kinase, plant-type, putative [*Ricinus communis*] | 2e-57 |
| LT21-1297 | JG293592 | 1 | 1207 | Transducin family protein / WD-40 repeat family protein [*Arabidopsis thaliana*] | 3e-86 |
| LT21-1500 | JG293593 | 1 | 1209 | Transducin family protein / WD-40 repeat family protein [*Arabidopsis thaliana*] | 4e-27 |
| LT21-4687 | JG293594 | 1 | 822 | Transducin family protein / WD-40 repeat family protein [*Arabidopsis thaliana*] | 2e-75 |
| LT21-1171 | JG293595 | 1 | 448 | Ubiquitin-protein ligase, putative [*Ricinus communis*] | 2e-32 |
| LT21-4405 | JG293596 | 1 | 1120 | Ubiquitin-protein ligase, putative [*Ricinus communis*] | 9e-120 |
| LT21-959 | JG293597 | 1 | 1148 | Ubiquitin-specific protease 24 [*Arabidopsis lyrata*] | 4e-13 |
| Contig 190 | JG293598 | 2 | 732 | Polyubiquitin [*Arabidopsis lyrata*] | 9e-73 |
| Contig 220 | JG293599 | 4 | 731 | Polyubiquitin containing 7 ubiquitin monomers [*Zea mays*] | 1e-78 |
| LT21-744 | JG293600 | 1 | 727 | Ras family GTP-binding protein [*Brassica oleracea*] | 1e-05 |
| LT21-4193 | JG293601 | 1 | 654 | Small GTP-binding protein [*Nicotiana plumbaginifolia*] | 2e-75 |
| LT21-1963 | JG293602 | 1 | 881 | Ras gtpase; Sigma-54 factor, interaction region [*Medicago truncatula*] | 6e-98 |
| LT21-4006 | JG293603 | 1 | 281 | Rab family gtpase [*Selaginella moellendorffii*] | 4e-22 |
| LT21-769 | JG293604 | 1 | 1178 | Rho gtpase activator, putative [*Ricinus communis*] | 3e-15 |
| LT21-1158 | JG293605 | 1 | 726 | Dynamin, putative [*Ricinus communis*] | 2e-50 |
| LT21-3099 | JG293606 | 1 | 279 | Dynamin, putative [*Ricinus communis*] | 6e-24 |
| LT21-265 | JG293607 | 1 | 626 | Parvulin-type peptidyl-prolyl cis/trans isomerase [*Lotus japonicus*] | 1e-46 |
| LT21-3416 | JG293608 | 1 | 1018 | Peptidylprolyl isomerase, putative [*Ricinus communis*] | 9e-86 |
| LT21-1435 | JG293609 | 1 | 1215 | Receptor for activated protein kinase C, putative [*Ricinus communis*] | 5e-105 |
| LT21-2104 | JG293610 | 1 | 1091 | Receptor protein kinase, putative [*Ricinus communis*] | 1e-40 |
| Contig 28 | JG293611 | 2 | 618 | Steroid binding protein, putative [*Ricinus communis*] | 1e-55 |
| Contig 339 | JG293612 | 2 | 1205 | Ran-binding protein 1 [*Cucumis melo*] | 4e-67 |
| Contig 353 | JG293613 | 2 | 1168 | Methyl-cpg binding [*Medicago truncatula*] | 1e-50 |
| LT21-3198 | JG293614 | 1 | 1136 | Rabgap/TBC domain-containing protein [*Arabidopsis lyrata*] | 4e-38 |
| Contig 335 | JG293615 | 3 | 1139 | Prenylated Rab acceptor protein, putative [*Ricinus communis*] | 8e-43 |
| LT21-3884 | JG293616 | 1 | 263 | Adenylate kinase [*Cucumis melo*] | 1e-25 |
| LT21-3688 | JG293617 | 1 | 930 | Seed calcium dependent protein kinase a [*Glycine max*] | 5e-77 |
| Contig 543 | JG293618 | 3 | 1048 | Lectin-like receptor kinase 7;2 [*Medicago truncatula*] | 5e-68 |
| Contig 602 | JG293619 | 2 | 1160 | Inositol pentakisphosphate 2-kinase [*Glycine max*] | 3e-60 |
| LT21-3887 | JG293620 | 1 | 673 | Diacylglycerol kinase, putative [*Arabidopsis thaliana*] | 2e-41 |
| Contig 382 | JG293621 | 2 | 1033 | Choline/ethanolamine kinase, putative [*Ricinus communis*] | 2e-59 |
| LT21-367 | JG293622 | 1 | 301 | CBL-interacting protein kinase 24 [*Populus trichocarpa*] | 8e-37 |
| LT21-3669 | JG293623 | 1 | 827 | Casein kinase, putative [*Ricinus communis*] | 5e-26 |
| LT21-3434 | JG293624 | 1 | 752 | Ankyrin-kinase [*Medicago truncatula*] | 2e-57 |
| LT21-3306 | JG293625 | 1 | 842 | TPR domain containing protein [*Zea mays*] | 8e-20 |
| Contig 118 | JG293626 | 3 | 907 | Short chain dehydrogenase [*Solanum tuberosum*] | 1e-93 |
| LT21-1423 | JG293627 | 1 | 1177 | SH3 domain-containing protein [*Trifolium repens*] | 2e-109 |
| LT21-3736 | JG293628 | 1 | 380 | PAS/LOV protein 1 [*Glycine max*] | 6e-09 |
| LT21-614 | JG293629 | 1 | 640 | IMP dehydrogenase/GMP reductase, related [*Medicago truncatula*] | 4e-19 |
| LT21-353 | JG293630 | 1 | 1051 | Histidine triad family protein [*Arabidopsis lyrata*] | 1e-55 |
| LT21-978 | JG293631 | 1 | 1175 | Gamma-glutamylcysteine synthetase precursor [*Pisum sativum*] | 4e-60 |
| LT21-166 | JG293632 | 1 | 822 | Exostosin family protein [*Arabidopsis lyrata* ] | 3e-82 |
| LT21-1179 | JG293633 | 1 | 1034 | Erythroblast macrophage protein emp, putative [*Ricinus communis*] | 2e-66 |
| LT21-2913 | JG293634 | 1 | 1145 | EREBP-4 like protein [*Arabidopsis thaliana*] | 8e-41 |
| LT21-2932 | JG293635 | 1 | 1118 | EF hand family protein [*Solanum demissum*] | 1e-43 |
| LT21-2489 | JG293636 | 1 | 1104 | Defective in cullin neddylation protein, putative [*Ricinus communis*] | 2e-62 |
| LT21-2444 | JG293637 | 1 | 807 | Chaperone dnak [*Medicago truncatula*] | 4e-69 |
| Contig 473 | JG293638 | 2 | 1188 | Auxin-responsive family protein [*Arabidopsis lyrata*] | 2e-61 |
| LT21-3810 | JG293639 | 1 | 751 | AKIN beta3 [*Medicago truncatula*] | 4e-31 |
| ***Membrane and transport*** **(101)** | | | | | |
| LT21-1535 | JG293640 | 1 | 1187 | Oligopeptide transporter OPT family [*Populus trichocarpa*] | 2e-111 |
| LT21-2220 | JG293641 | 1 | 797 | Oligopeptide transporter, putative [*Ricinus communis*] | 2e-91 |
| LT21-1338 | JG293642 | 1 | 1085 | Oligopeptide transporter, putative [*Ricinus communis*] | 2e-67 |
| Contig 56 | JG293643 | 2 | 1122 | Phloem specific protein [*Pisum sativum*] | 2e-37 |
| LT21-261 | JG293644 | 1 | 654 | Phloem specific protein [*Pisum sativum*] | 4e-10 |
| LT21-3660 | JG293645 | 1 | 630 | Synaptobrevin-related family protein [*Arabidopsis thaliana*] | 5e-29 |
| LT21-1967 | JG293646 | 1 | 804 | Synaptosomal associated protein, putative [*Ricinus communis*] | 3e-10 |
| LT21-4063 | JG293647 | 1 | 539 | Syntaxin, putative [*Ricinus communis*] | 2e-56 |
| LT21-4187 | JG293648 | 1 | 423 | Syntaxin, putative [*Ricinus communis*] | 3e-32 |
| LT21-4103 | JG293649 | 1 | 795 | Vesicle-associated membrane protein 7B [*Arabidopsis lyrata*] | 3e-20 |
| LT21-1190 | JG293650 | 1 | 841 | Vesicle-associated membrane protein, putative [*Ricinus communis*] | 3e-93 |
| LT21-3084 | JG293651 | 1 | 1190 | Vesicle-associated membrane protein, putative [*Ricinus communis*] | 5e-102 |
| LT21-2602 | JG293652 | 1 | 373 | Vesicle-associated membrane protein, putative [*Ricinus communis*] | 5e-42 |
| Contig 47 | JG293653 | 2 | 1100 | Vesicle-associated membrane protein-related [*Arabidopsis thaliana*] | 2e-17 |
| Contig 64 | JG293654 | 2 | 1100 | Vesicle-associated membrane protein-related [*Arabidopsis thaliana*] | 2e-17 |
| LT21-1709 | JG293655 | 1 | 489 | VHS and GAT domain protein [*Glycine max*] | 2e-13 |
| LT21-4093 | JG293656 | 1 | 386 | VHS and GAT domain protein [*Glycine max*] | 6e-52 |
| LT21-1570 | JG293657 | 1 | 582 | Nuclear transport factor, putative [*Ricinus communis*] | 8e-55 |
| LT21-2070 | JG293658 | 1 | 581 | Nuclear transport factor, putative [*Ricinus communis*] | 2e-48 |
| LT21-906 | JG293659 | 1 | 1169 | Adenine nucleotide translocator [*Lupinus albus*] | 4e-96 |
| LT21-180 | JG293660 | 1 | 923 | ATP-dependent transporter, putative [*Ricinus communis*] | 3e-28 |
| LT21-252 | JG293661 | 1 | 600 | ATP-dependent transporter, putative [*Ricinus communis*] | 5e-09 |
| LT21-270 | JG293662 | 1 | 1104 | ATP-dependent transporter, putative [*Ricinus communis*] | 4e-05 |
| LT21-660 | JG293663 | 1 | 1200 | ATP/ADP transporter [*Populus trichocarpa*] | 1e-54 |
| LT21-1332 | JG293664 | 1 | 842 | ATP-binding cassette transporter, putative [*Ricinus communis*] | 2e-09 |
| LT21-238 | JG293665 | 1 | 894 | ATP-binding cassette transporter, putative [*Ricinus communis*] | 3e-13 |
| Contig 463 | JG293666 | 2 | 889 | ABC transporter homolog [*Populus nigra*] | 2e-138 |
| LT21-4878 | JG293667 | 1 | 378 | ABC1 family protein [*Arabidopsis thaliana*] | 4e-17 |
| LT21-4894 | JG293668 | 1 | 807 | Al-induced protein [*Jatropha curcas*] | 3e-59 |
| Contig 619 | JG293669 | 2 | 1141 | Al-induced protein [*Gossypium hirsutum*] | 8e-96 |
| LT21-3753 | JG293670 | 1 | 553 | Al-induced protein [*Gossypium hirsutum*] | 5e-45 |
| LT21-2825 | JG293671 | 1 | 1021 | Aquaporin sip2.1, putative [*Ricinus communis*] | 2e-73 |
| LT21-2928 | JG293672 | 1 | 1113 | Aquaporin, MIP family, SIP subfamily [*Populus trichocarpa*] | 2e-66 |
| LT21-1873 | JG293673 | 1 | 1174 | Aquaporin-like transmembrane channel protein [*Galega orientalis*] | 7e-143 |
| LT21-4202 | JG293674 | 1 | 491 | Potassium channel beta, putative [*Ricinus communis*] | 1e-32 |
| LT21-1922 | JG293675 | 1 | 1120 | Potassium transporter, putative [*Ricinus communis*] | 1e-94 |
| LT21-2112 | JG293676 | 1 | 1184 | ADP,ATP carrier protein, putative [*Ricinus communis*] | 6e-96 |
| LT21-3838 | JG293677 | 1 | 785 | Putative ADP,ATP carrier-like protein [*Trifolium pratense*] | 6e-38 |
| LT21-1357 | JG293678 | 1 | 1154 | Cation efflux protein/ zinc transporter, putative [*Ricinus communis*] | 3e-35 |
| LT21-4568 | JG293679 | 1 | 836 | Cation-transporting atpase plant, putative [*Ricinus communis*] | 2e-23 |
| LT21-2149 | JG293680 | 1 | 533 | Sugar transporter, putative [*Ricinus communis*] | 2e-80 |
| LT21-2437 | JG293681 | 1 | 488 | Sugar transporter, putative [*Ricinus communis*] | 7e-16 |
| LT21-3867 | JG293682 | 1 | 921 | UDP-galactose transporter homolog 1, related [*Medicago truncatula*] | 8e-08 |
| LT21-2277 | JG293683 | 1 | 1195 | Hexose transporter [*Solanum lycopersicum*] | 4e-95 |
| Contig 582 | JG293684 | 2 | 1141 | General substrate transporter [*Medicago truncatula*] | 6e-109 |
| LT21-4022 | JG293685 | 1 | 1241 | Integral membrane transporter family protein [*Arabidopsis thaliana*] | 6e-83 |
| LT21-2034 | JG293686 | 1 | 1191 | Metal transporter Nramp3 [*Arabidopsis thaliana*] | 2e-41 |
| Contig 645 | JG293687 | 2 | 1194 | Phosphate transporter [*Glycine max*] | 5e-78 |
| LT21-1806 | JG293688 | 1 | 891 | Putative transporter-like protein [*Trifolium pratense*] | 3e-77 |
| LT21-1673 | JG293689 | 1 | 1236 | Protein transport protein SEC61 subunit gamma [*Jatropha curcas*] | 1e-18 |
| LT21-1395 | JG293690 | 1 | 764 | Transporter, putative [*Ricinus communis*] | 4e-35 |
| LT21-867 | JG293691 | 1 | 1200 | Sec61 transport protein [*Populus trichocarpa*] | 5e-98 |
| LT21-3988 | JG293692 | 1 | 1180 | Transmembrane emp24 domain-containing protein 10 precursor, putative [*Ricinus communis*] | 1e-66 |
| LT21-1758 | JG293693 | 1 | 1138 | Transmembrane protein 14, putative [*Ricinus communis*] | 2e-35 |
| LT21-4673 | JG293694 | 1 | 1154 | Transmembrane protein TPARL, putative [*Ricinus communis*] | 8e-36 |
| Contig 168 | JG293695 | 2 | 1051 | Ankyrin-repeat membrane protein, IGN1 [*Lotus japonicus*] | 3e-60 |
| LT21-4049 | JG293696 | 1 | 753 | Membrane protein [*Arabidopsis thaliana*] | 1e-19 |
| LT21-4534 | JG293697 | 1 | 1117 | Putative endomembrane protein precursor [*Medicago sativa*] | 3e-109 |
| Contig 334 | JG293698 | 2 | 1226 | Secretory carrier membrane protein, putative [*Ricinus communis*] | 8e-78 |
| LT21-3905 | JG293699 | 1 | 402 | Exocyst complex component, putative [*Ricinus communis*] | 2e-44 |
| LT21-4349 | JG293700 | 1 | 502 | Exocyst complex component sec3, putative [*Ricinus communis*] | 2e-27 |
| LT21-3297 | JG293701 | 1 | 665 | Vacuolar (H+)-atpase G subunit; KH, prokaryotic type [*Medicago truncatula*] | 8e-23 |
| LT21-501 | JG293702 | 1 | 395 | Vacuolar ATP synthase subunit F [*Zea mays*] | 3e-24 |
| LT21-3997 | JG293703 | 1 | 277 | V-atpase subunit C [*Medicago truncatula*] | 1e-08 |
| LT21-2995 | JG293704 | 1 | 1215 | Acyl-ACP thioesterase [*Glycine max*] | 1e-121 |
| LT21-3063 | JG293705 | 1 | 406 | Acyl-coa-binding protein [*Zea mays*] | 6e-13 |
| LT21-1871 | JG293706 | 1 | 641 | Mitochondrial substrate carrier family protein [*Arabidopsis lyrata* ssp. *lyrata*] | 5e-08 |
| LT21-1247 | JG293707 | 1 | 924 | Putative mitochondrial dicarboxylate carrier protein [*Trifolium pratense*] | 2e-62 |
| LT21-1082 | JG293708 | 1 | 764 | Adapter-related protein complex 1 beta 1 subunit, putative, expressed [*Oryza sativa*] | 1e-59 |
| LT21-1030 | JG293709 | 1 | 1106 | Adaptin family protein [*Arabidopsis thaliana*] | 2e-72 |
| LT21-4903 | JG293710 | 1 | 973 | Nodule-enhanced sucrose synthase [*Pisum sativum*] | 3e-133 |
| Contig 78 | JG293711 | 6 | 1220 | Oxygen-evolving enhancer protein 1, chloroplastic [*Arabidopsis thaliana*] | 3e-95 |
| LT21-1716 | JG293712 | 1 | 735 | Type IIB calcium atpase [*Medicago truncatula*] | 2e-54 |
| LT21-2988 | JG293713 | 1 | 1096 | Calcium ion binding protein, putative [*Ricinus communis*] | 1e-57 |
| LT21-2205 | JG293714 | 1 | 778 | Transferase, transferring glycosyl groups, putative [*Ricinus communis*] | 7e-105 |
| LT21-3550 | JG293715 | 1 | 1187 | Sterol-C5(6)-desaturase homolog [*Nicotiana tabacum*] | 6e-100 |
| LT21-1453 | JG293716 | 1 | 1022 | Sec-independent protein translocase protein tatc, putative [*Ricinus communis*] | 7e-47 |
| LT21-1667 | JG293717 | 1 | 1205 | Oleate desaturase [*Caragana korshinskii* var. Intermedia] | 1e-132 |
| LT21-2824 | JG293718 | 1 | 1097 | ARF gtpase activator, putative [*Ricinus communis*] | 1e-61 |
| Contig 402 | JG293719 | 2 | 1108 | Allantoin permease [*Phaseolus vulgaris*] | 6e-80 |
| LT21-4663 | JG293720 | 1 | 1208 | Alpha-soluble NSF attachment protein [*Gossypium hirsutum*] | 1e-103 |
| LT21-4005 | JG293721 | 1 | 583 | Armadillo/beta-catenin repeat family protein [*Arabidopsis thaliana*] | 4e-45 |
| LT21-2063 | JG293722 | 1 | 993 | Auxin-induced protein 5NG4, putative [*Ricinus communis*] | 5e-46 |
| LT21-1143 | JG293723 | 1 | 1080 | Band 7 family protein [*Arabidopsis thaliana*] | 2e-101 |
| LT21-2383 | JG293724 | 1 | 890 | Charged multivesicular body protein 2b-B, putative [*Ricinus communis*] | 1e-45 |
| LT21-1209 | JG293725 | 1 | 990 | Chmp1, putative [*Ricinus communis*] | 3e-35 |
| LT21-47 | JG293726 | 1 | 828 | Clathrin heavy chain, putative [*Ricinus communis*] | 3e-47 |
| Contig 98 | JG293727 | 3 | 461 | Coatomer beta subunit, putative [*Ricinus communis*] | 5e-12 |
| LT21-3538 | JG293728 | 1 | 1018 | Fiddlehead-like protein [*Pisum sativum*] | 1e-102 |
| Contig 163 | JG293729 | 2 | 688 | Hydrophobic protein LTI6B [*Zea mays*] | 2e-04 |
| LT21-1543 | JG293730 | 1 | 651 | Multidrug resistance pump, putative [*Ricinus communis*] | 5e-33 |
| LT21-3217 | JG293731 | 1 | 1207 | Novel plant snare, putative [*Ricinus communis*] | 1e-67 |
| LT21-745 | JG293732 | 1 | 1191 | Patellin-3, putative [*Ricinus communis*] | 1e-83 |
| LT21-108 | JG293733 | 1 | 1176 | Prefoldin [*Medicago truncatula*] | 4e-55 |
| Contig 34 | JG293734 | 4 | 1159 | Prenylated Rab acceptor protein, putative [*Ricinus communis*] | 2e-48 |
| LT21-2126 | JG293735 | 1 | 1192 | Putative extracellular dermal glycoprotein [*Cicer arietinum*] | 2e-64 |
| Contig 412 | JG293736 | 2 | 534 | SEC14 cytosolic factor / phosphoglyceride transfer family protein [*Ipomoea nil*] | 1e-30 |
| LT21-2129 | JG293737 | 1 | 365 | Sec61beta family protein [*Arabidopsis thaliana*] | 1e-14 |
| LT21-2570 | JG293738 | 1 | 424 | Vf14-3-3c protein [*Vicia faba*] | 3e-51 |
| LT21-2979 | JG293739 | 1 | 1197 | Transport inhibitor response 1 protein, putative [*Ricinus communis*] | 5e-110 |
| ***Stress and defense*** **(387)** | | | | | |
| Contig 302 | JG293740 | 2 | 1098 | 17.9 kda heat shock protein (hsp17.9) [*Pisum sativum*] | 2e-51 |
| Contig 396 | JG293741 | 2 | 1108 | 17.9 kda heat shock protein (hsp17.9) [*Pisum sativum*] | 4e-60 |
| Contig 397 | JG293742 | 2 | 1162 | 17.9 kda heat shock protein (hsp17.9) [*Pisum sativum*] | 4e-56 |
| Contig 423 | JG293743 | 3 | 1213 | 17.9 kda heat shock protein (hsp17.9) [*Pisum sativum*] | 2e-47 |
| Contig 540 | JG293744 | 2 | 1035 | 17.9 kda heat shock protein (hsp17.9) [*Pisum sativum*] | 2e-57 |
| LT21-1662 | JG293745 | 1 | 1046 | 17.9 kda heat shock protein (hsp17.9) [*Pisum sativum*] | 1e-63 |
| LT21-1807 | JG293746 | 1 | 767 | 17.9 kda heat shock protein (hsp17.9) [*Pisum sativum*] | 2e-56 |
| LT21-2344 | JG293747 | 1 | 419 | 17.9 kda heat shock protein (hsp17.9) [*Pisum sativum*] | 4e-05 |
| LT21-1225 | JG293748 | 1 | 778 | Aldo/keto reductase [*Medicago truncatula*] | 2e-76 |
| LT21-875 | JG293749 | 1 | 951 | Aldo/keto reductase [*Medicago truncatula*] | 2e-98 |
| LT21-7 | JG293750 | 1 | 1135 | Aldo/keto reductase [*Medicago truncatula*] | 8e-130 |
| Contig 643 | JG293751 | 2 | 1144 | Small heat-shock protein [*Pisum sativum*] | 3e-49 |
| LT21-2645 | JG293752 | 1 | 781 | Small heat-shock protein [*Pisum sativum*] | 1e-25 |
| Contig 67 | JG293753 | 2 | 880 | Small heat shock protein [*Retama raetam*] | 4e-58 |
| Contig 306 | JG293754 | 2 | 1240 | Small heat-shock protein [*Pisum sativum*] | 8e-40 |
| LT21-3954 | JG293755 | 1 | 1214 | Small heat-shock protein, putative [*Ricinus communis*] | 3e-34 |
| Contig 495 | JG293756 | 2 | 1097 | Small molecular heat shock protein 10 [*Nelumbo nucifera*] | 1e-27 |
| LT21-4747 | JG293757 | 1 | 427 | Small molecular heat shock protein 10 [*Nelumbo nucifera*] | 2e-18 |
| Contig 147 | JG293758 | 4 | 743 | CII small heat shock protein 1 [*Prunus salicina*] | 4e-62 |
| LT21-4398 | JG293759 | 1 | 542 | 70 kd heats hock protein [*Medicago sativa*] | 1e-35 |
| Contig 184 | JG293760 | 2 | 1209 | Chloroplast small heat shock protein 1 [*Potentilla discolor*] | 2e-27 |
| LT21-2107 | JG293761 | 1 | 487 | Putative HEAT SHOCK PROTEIN 81-2 [*Trifolium pratense*] | 2e-44 |
| LT21-3023 | JG293762 | 1 | 793 | DNAJ heat shock family protein [*Arabidopsis lyrata* ssp. *lyrata*] | 4e-39 |
| Contig 523 | JG293763 | 2 | 665 | Ankyrin repeat-containing protein, putative [*Ricinus communis*] | 4e-53 |
| LT21-1713 | JG293764 | 1 | 552 | Ankyrin repeat-containing protein, putative [*Ricinus communis*] | 3e-23 |
| LT21-1109 | JG293765 | 1 | 1176 | Aspartic proteinase 2 [*Glycine max*] | 7e-137 |
| LT21-448 | JG293766 | 1 | 925 | Aspartic proteinase nepenthesin-1 precursor, putative [*Ricinus communis*] | 4e-61 |
| Contig 336 | JG293767 | 2 | 1227 | ATP-dependent Clp protease proteolytic subunit 2 [*Zea mays*] | 4e-69 |
| LT21-1487 | JG293768 | 1 | 1148 | ATP-dependent Clp protease proteolytic subunit, putative [*Ricinus communi*s] | 6e-96 |
| LT21-692 | JG293769 | 1 | 1122 | ATP-dependent Clp protease proteolytic subunit, putative [*Ricinus communis*] | 8e-89 |
| Contig 652 | JG293770 | 2 | 1158 | ATP-binding cassette transporter, putative [*Ricinus communis*] | 2e-94 |
| Contig 141 | JG293771 | 6 | 1004 | Bax inhibitor [*Capsicum annuum*] | 7e-71 |
| LT21-1044 | JG293772 | 1 | 489 | Bax inhibitor, putative [*Ricinus communis*] | 1e-19 |
| LT21-3216 | JG293773 | 1 | 419 | Bax inhibitor, putative [*Ricinus communis*] | 2e-34 |
| LT21-887 | JG293774 | 1 | 751 | Beta-1,3 glucanase [*Pisum sativum*] | 3e-62 |
| Contig 110 | JG293775 | 4 | 711 | Beta-1,3-glucanase [*Sesbania rostrata*] | 3e-59 |
| LT21-3741 | JG293776 | 1 | 183 | Beta-1,3-glucanase 3 [*Glycine max*] | 2e-07 |
| LT21-766 | JG293777 | 1 | 1037 | 1,3-beta-glucan synthase [*Malus x domestica*] | 3e-91 |
| LT21-3381 | JG293778 | 1 | 510 | Acidic class II 1,3-beta-glucanase precursor [*Solanum tuberosum*] | 3e-17 |
| Contig 517 | JG293779 | 2 | 1161 | Acidic class II 1,3-beta-glucanase precursor [*Solanum tuberosum*] | 3e-39 |
| LT21-4165 | JG293780 | 1 | 360 | Glucan synthase-like 7 [*Hordeum vulgare*] | 7e-21 |
| Contig 503 | JG293781 | 2 | 1176 | Glucosyltransferase-13 [*Vigna angularis*] | 1e-48 |
| LT21-694 | JG293782 | 1 | 766 | Glucosyltransferase-6 [*Vigna angularis*] | 1e-28 |
| LT21-2050 | JG293783 | 1 | 1149 | Endo-1,3-1,4-beta-d-glucanase, putative [*Ricinus communis*] | 3e-76 |
| Contig 72 | JG293784 | 23 | 1179 | Endo-1,3-beta-glucanase [*Glycine max*] | 5e-86 |
| LT21-3130 | JG293785 | 1 | 344 | Endo-1,3-beta-glucanase [*Glycine max*] | 5e-05 |
| Contig 165 | JG293786 | 4 | 937 | Endo-1,3-1,4-beta-d-glucanase, putative [*Ricinus communis*] | 1e-73 |
| Contig 636 | JG293787 | 2 | 1145 | Endo-1,3-1,4-beta-d-glucanase, putative [*Ricinus communis*] | 1e-73 |
| Contig 389 | JG293788 | 2 | 1156 | Endoglucanase [*Nectria ipomoeae*] | 2e-84 |
| Contig 661 | JG293789 | 2 | 1123 | Chaperone protein dnaj, putative [*Ricinus communis*] | 6e-87 |
| LT21-2814 | JG293790 | 1 | 1174 | Chaperone protein dnaj, putative [*Ricinus communis*] | 4e-77 |
| Contig 457 | JG293791 | 2 | 681 | Chaperone protein dnaj 11, chloroplast precursor, putative [*Ricinus communis*] | 4e-31 |
| LT21-396 | JG293792 | 1 | 883 | Dnaj-like protein [*Medicago sativa*] | 3e-73 |
| LT21-3898 | JG293793 | 1 | 741 | Chitinase [*Trifolium repens*] | 7e-28 |
| LT21-4071 | JG293794 | 1 | 839 | Chitinase [*Trifolium repens*] | 6e-39 |
| LT21-3868 | JG293795 | 1 | 1061 | Class Ia chitinase [*Galega orientalis*] | 7e-107 |
| LT21-2217 | JG293796 | 1 | 699 | Class Ib chitinase [*Galega orientalis*] | 4e-101 |
| LT21-4043 | JG293797 | 1 | 866 | Class III chitinase [*Lupinus albus*] | 1e-61 |
| LT21-4804 | JG293798 | 1 | 1058 | Cold responsive protein TRVSP [*Trifolium repens*] | 1e-27 |
| Contig 282 | JG293799 | 2 | 1104 | Cold-regulated protein [*Glycine max*] | 9e-27 |
| Contig 441 | JG293800 | 3 | 1210 | Cold-regulated protein [*Glycine max*] | 1e-60 |
| LT21-1483 | JG293801 | 1 | 756 | Copine, putative [*Ricinus communis*] | 1e-15 |
| LT21-3235 | JG293802 | 1 | 1141 | Copine, putative [*Ricinus communis*] | 2e-43 |
| LT21-3000 | JG293803 | 1 | 628 | Cystatin [*Medicago sativa*] | 1e-32 |
| Contig 309 | JG293804 | 2 | 420 | Cystatin-like protein [*Arabidopsis thaliana*] | 3e-21 |
| Contig 549 | JG293805 | 2 | 1148 | Cysteine protease [*Pisum sativum*] | 2e-115 |
| LT21-4453 | JG293806 | 1 | 607 | Cysteine proteinase inhibitor [*Populus tomentosa*] | 1e-37 |
| LT21-1927 | JG293807 | 1 | 1158 | Cysteine-type peptidase, putative [*Ricinus communis*] | 1e-19 |
| LT21-1256 | JG293808 | 1 | 728 | Cytochome b5 [*Olea europaea*] | 1e-62 |
| LT21-1751 | JG293809 | 1 | 675 | Cytochrome B5 isoform 1, putative [*Ricinus communis*] | 9e-41 |
| LT21-1696 | JG293810 | 1 | 606 | Cytochrome c, monohaem [*Medicago truncatula*] | 4e-43 |
| Contig 322 | JG293811 | 2 | 1013 | Cytochrome P450 [*Cicer arietinum*] | 4e-88 |
| LT21-4722 | JG293812 | 1 | 895 | Cytochrome P450 [*Glycyrrhiza echinata*] | 2e-102 |
| LT21-112 | JG293813 | 1 | 1131 | Cytochrome P450 [*Populus trichocarpa*] | 5e-63 |
| LT21-1640 | JG293814 | 1 | 333 | Cytochrome P450 [*Populus trichocarpa*] | 3e-13 |
| LT21-2785 | JG293815 | 1 | 1149 | Cytochrome P450 [*Populus trichocarpa*] | 2e-66 |
| LT21-4983 | JG293816 | 1 | 1037 | Cytochrome P450 [*Populus trichocarpa*] | 1e-64 |
| LT21-95 | JG293817 | 1 | 799 | Cytochrome P450 [*Populus trichocarpa*] | 2e-82 |
| LT21-2484 | JG293818 | 1 | 349 | Cytochrome P450 monooxygenase CYP710A15 [*Medicago truncatula*] | 4e-43 |
| Contig 362 | JG293819 | 2 | 767 | Cytochrome P450 monooxygenase CYP76O2 [*Glycine max*] | 2e-42 |
| LT21-1074 | JG293820 | 1 | 451 | Cytochrome P450 monooxygenase CYP76O2 [*Glycine max*] | 3e-30 |
| Contig 340 | JG293821 | 2 | 1235 | Cytochrome P450, putative [*Ricinus communis*] | 3e-69 |
| LT21-3005 | JG293822 | 1 | 738 | Cytosolic heat shock protein 90.1 [*Dactylis glomerata*] | 2e-68 |
| LT21-2237 | JG293823 | 1 | 496 | Defensin-like protein [*Vicia faba*] | 1e-34 |
| LT21-3856 | JG293824 | 1 | 1070 | Dehydration responsive element-binding protein 3 [*Glycine max*] | 5e-58 |
| LT21-2229 | JG293825 | 1 | 488 | Dehydration responsive protein [*Glycine max*] | 1e-69 |
| Contig 569 | JG293826 | 2 | 1166 | Dehydration-responsive element binding protein 3 [*Glycine max*] | 5e-18 |
| LT21-194 | JG293827 | 1 | 963 | Dehydration-responsive element binding protein 3 [*Glycine max*] | 7e-47 |
| Contig 614 | JG293828 | 2 | 596 | Dehydrin [*Pistacia vera*] | 1e-05 |
| LT21-200 | JG293829 | 1 | 1061 | Dehydrin a [*Vicia monantha*] | 4e-30 |
| Contig 405 | JG293830 | 4 | 1181 | Dehydrin-cognate [*Pisum sativum*] | 3e-40 |
| LT21-440 | JG293831 | 1 | 525 | Disease resistance protein/LRR protein-related protein [*Glycine max*] | 2e-46 |
| Contig 139 | JG293832 | 2 | 1195 | Disease-resistance protein [*Glycine max*] | 2e-68 |
| LT21-967 | JG293833 | 1 | 1055 | Verticillium wilt disease resistance protein precursor [*Solanum torvum*] | 4e-35 |
| LT21-2587 | JG293834 | 1 | 622 | Multidrug resistance protein ABC transporter family [*Populus trichocarpa*] | 2e-50 |
| LT21-1904 | JG293835 | 1 | 915 | Natural resistance-associated macrophage protein, putative [*Ricinus communis*] | 1e-73 |
| LT21-1990 | JG293836 | 1 | 1170 | NB-LRR type disease resistance protein Rps1-k-1 [*Glycine max*] | 3e-73 |
| Contig 65 | JG293837 | 9 | 1216 | Nematode resistance HS1pro1 protein [*Glycine max*] | 1e-70 |
| LT21-4786 | JG293838 | 1 | 661 | Grave disease carrier protein, putative [*Ricinus communis*] | 6e-49 |
| LT21-2230 | JG293839 | 1 | 1134 | Endoplasmic reticulum HSC70-cognate binding protein precursor [*Glycine max*] | 2e-103 |
| LT21-1856 | JG293840 | 1 | 837 | Ethylene response factor ERF1 [*Arabidopsis thaliana*] | 5e-08 |
| Contig 349 | JG293841 | 2 | 1087 | Ethylene-responsive transciptional coactivator-like protein [*Retama raetam*] | 2e-54 |
| Contig 346 | JG293842 | 3 | 1055 | Contains similarity to ethylene responsive element binding factor [*Arabidopsis thaliana*] | 3e-26 |
| LT21-3171 | JG293843 | 1 | 1163 | Ferritin [*Medicago sativa* ssp. *falcata*] | 1e-75 |
| Contig 471 | JG293844 | 3 | 1178 | Ferritin [*Medicago sativa*] | 1e-74 |
| LT21-3638 | JG293845 | 1 | 478 | Gamma-glutamylcysteine synthetase precursor [*Pisum sativum*] | 1e-06 |
| LT21-4224 | JG293846 | 1 | 502 | Gamma-glutamylcysteine synthetase precursor [*Pisum sativum*] | 2e-34 |
| LT21-1207 | JG293847 | 1 | 908 | Glutathione peroxidase 1 [*Lotus japonicus*] | 4e-81 |
| LT21-4001 | JG293848 | 1 | 713 | Glutathione peroxidase 1 [*Lotus japonicus*] | 7e-62 |
| LT21-4874 | JG293849 | 1 | 322 | Glutathione S-transferase [*Arachis diogoi*] | 2e-10 |
| Contig 82 | JG293850 | 5 | 982 | Glutathione S-transferase [*Caragana korshinskii*] | 6e-92 |
| LT21-3273 | JG293851 | 1 | 1007 | Glutathione S-transferase [*Caragana korshinskii*] | 6e-81 |
| LT21-3714 | JG293852 | 1 | 1091 | Glutathione S-transferase [*Citrus sinensis*] | 3e-15 |
| Contig 326 | JG293853 | 5 | 1214 | Glutathione S-transferase [*Pisum sativum*] | 2e-107 |
| LT21-4250 | JG293854 | 1 | 1020 | Glutathione S-transferase [*Pisum sativum*] | 2e-12 |
| Contig 648 | JG293855 | 2 | 902 | Glutathione S-transferase [*Vitis vinifera*] | 1e-77 |
| LT21-4344 | JG293856 | 1 | 1032 | Glutathione S-transferase [*Vitis vinifera*] | 3e-54 |
| LT21-1886 | JG293857 | 1 | 1237 | Glutathione S-transferase GST 13 [*Glycine max*] | 3e-37 |
| LT21-3822 | JG293858 | 1 | 502 | Glutathione S-transferase GST 17 [*Glycine max*] | 4e-44 |
| LT21-1893 | JG293859 | 1 | 1023 | Glutathione S-transferase GST 18 [*Glycine max*] | 3e-42 |
| LT21-344 | JG293860 | 1 | 1064 | Glutathione S-transferase GST 24 [*Glycine max*] | 7e-61 |
| Contig 592 | JG293861 | 2 | 660 | Glutathione S-transferase GST 25 [*Glycine max*] | 9e-37 |
| Contig 66 | JG293862 | 4 | 865 | Glutathione S-transferase GST 5 [*Glycine max*] | 5e-39 |
| LT21-2844 | JG293863 | 1 | 972 | Glutathione S-transferase GST 7 [*Glycine max*] | 3e-40 |
| LT21-3249 | JG293864 | 1 | 816 | Glutathione S-transferase GST 7 [*Glycine max*] | 5e-77 |
| LT21-2090 | JG293867 | 1 | 842 | Glutathione S-transferase, C-terminal; Thioredoxin-like fold [*Medicago truncatula*] | 3e-55 |
| LT21-3055 | JG293868 | 1 | 736 | Glutathione S-transferase, C-terminal; Thioredoxin-like fold [*Medicago truncatula*] | 3e-60 |
| LT21-3065 | JG293869 | 1 | 1207 | Glutathione synthetase precursor [*Pisum sativum*] | 2e-91 |
| Contig 83 | JG293870 | 3 | 878 | Recname: Full=Probable glutathione S-transferase; | 3e-68 |
| LT21-4253 | JG293871 | 1 | 471 | Tau class glutathione transferase GSTU43 [*Populus trichocarpa*] | 9e-35 |
| Contig 206 | JG293872 | 2 | 1078 | Glycoside hydrolase, family 17 [*Medicago truncatula*] | 4e-122 |
| LT21-20 | JG293873 | 1 | 1107 | Glycoside hydrolase, family 17 [*Medicago truncatula*] | 1e-06 |
| LT21-1926 | JG293874 | 1 | 1074 | Heat- and acid-stable phosphoprotein [*Zea mays*] | 3e-42 |
| LT21-3643 | JG293875 | 1 | 1148 | Heat shock cognate 70 kda protein [*Phytolacca acinosa*] | 5e-75 |
| LT21-2399 | JG293876 | 1 | 1204 | Heat shock factor protein HSF30, putative [*Ricinus communis*] | 5e-34 |
| LT21-3248 | JG293877 | 1 | 541 | Heat shock factor protein, putative [*Ricinus communis*] | 3e-16 |
| Contig 197 | JG293878 | 10 | 1152 | Heat shock protein [*Ammopiptanthus mongolicus*] | 8e-61 |
| LT21-3716 | JG293879 | 1 | 930 | Heat shock protein [*Glycine max*] | 9e-85 |
| LT21-4824 | JG293880 | 1 | 1216 | Heat shock protein [*Glycine max*] | 9e-06 |
| Contig 22 | JG293881 | 8 | 709 | Heat shock protein [*Pisum sativum*] | 1e-63 |
| Contig 48 | JG293882 | 4 | 705 | Heat shock protein [*Pisum sativum*] | 2e-63 |
| Contig 149 | JG293883 | 2 | 688 | Heat shock protein [*Pisum sativum*] | 6e-57 |
| Contig 207 | JG293884 | 4 | 719 | Heat shock protein [*Pisum sativum*] | 2e-56 |
| Contig 249 | JG293885 | 5 | 1156 | Heat shock protein [*Pisum sativum*] | 6e-62 |
| Contig 265 | JG293886 | 6 | 1199 | Heat shock protein [*Pisum sativum*] | 1e-49 |
| Contig 287 | JG293887 | 4 | 675 | Heat shock protein [*Pisum sativum*] | 2e-61 |
| Contig 351 | JG293888 | 2 | 1150 | Heat shock protein [*Pisum sativum*] | 5e-64 |
| Contig 374 | JG293889 | 4 | 1166 | Heat shock protein [*Pisum sativum*] | 9e-66 |
| LT21-2164 | JG293890 | 1 | 1140 | Heat shock protein [*Pisum sativum*] | 1e-61 |
| LT21-3599 | JG293891 | 1 | 740 | Heat shock protein [*Pisum sativum*] | 9e-56 |
| LT21-58 | JG293892 | 1 | 709 | Heat shock protein [*Pisum sativum*] | 2e-68 |
| LT21-596 | JG293893 | 1 | 1004 | Heat shock protein [*Pisum sativum*] | 2e-50 |
| LT21-1529 | JG293894 | 1 | 1026 | Heat shock protein 101 [*Arabidopsis thaliana*] | 2e-80 |
| Contig 73 | JG293895 | 5 | 745 | Heat shock protein 17.5 cytosolic class II [*Jatropha curcas*] | 2e-53 |
| Contig 298 | JG293896 | 3 | 699 | Heat shock protein 17.5 cytosolic class II [*Jatropha curcas*] | 2e-28 |
| LT21-1275 | JG293897 | 1 | 1210 | Heat shock protein 70 (HSP70)-interacting protein, putative [*Ricinus communis*] | 3e-63 |
| LT21-2487 | JG293898 | 1 | 560 | Heat shock protein 70kd, putative [*Ricinus communis*] | 1e-50 |
| LT21-2518 | JG293899 | 1 | 181 | Heat shock protein 80 [*Triticum aestivum*] | 0.020 |
| LT21-1690 | JG293900 | 1 | 505 | Heat shock protein 81-4 [*Arabidopsis lyrata* ssp. *lyrata*] | 2e-13 |
| LT21-2770 | JG293901 | 1 | 693 | Heat shock protein 81-4 [*Arabidopsis lyrata* ssp. *lyrata*] | 1e-36 |
| LT21-3568 | JG293902 | 1 | 1103 | Heat shock protein 90 [*Triticum aestivum*] | 3e-101 |
| LT21-558 | JG293903 | 1 | 1138 | Heat shock protein binding [*Arabidopsis thaliana*] | 3e-63 |
| LT21-1567 | JG293904 | 1 | 1135 | Heat shock protein dnaj [*Medicago truncatula*] | 3e-82 |
| LT21-330 | JG293905 | 1 | 1147 | Heat shock protein dnaj [*Medicago truncatula*] | 6e-110 |
| Contig 50 | JG293906 | 2 | 1128 | Heat shock protein hsp70 | 5e-111 |
| LT21-2088 | JG293907 | 1 | 1219 | Heat shock protein Hsp70 [*Medicago truncatula*] | 1e-104 |
| LT21-1296 | JG293908 | 1 | 1237 | Heat shock protein, putative [*Ricinus communis*] | 2e-105 |
| LT21-2302 | JG293909 | 1 | 1167 | Heat shock protein, putative [*Ricinus communis*] | 2e-91 |
| LT21-3003 | JG293910 | 1 | 727 | Heat shock protein, putative [*Ricinus communis*] | 9e-32 |
| LT21-430 | JG293911 | 1 | 576 | Heat shock protein, putative [*Ricinus communis*] | 4e-64 |
| LT21-4120 | JG293912 | 1 | 460 | Heat shock transcription factor 34 [*Glycine max*] | 6e-15 |
| LT21-3819 | JG293913 | 1 | 641 | Heat-shock protein [*Arabidopsis thaliana*] | 2e-33 |
| Contig 159 | JG293914 | 2 | 736 | Heat-shock protein, putative [*Ricinus communis*] | 1e-35 |
| Contig 283 | JG293915 | 3 | 644 | Heat-shock protein, putative [*Ricinus communis*] | 3e-39 |
| LT21-4702 | JG293916 | 1 | 663 | Heat-shock protein, putative [*Ricinus communis*] | 9e-57 |
| Contig 224 | JG293917 | 3 | 1215 | Hsp22.5 [*Glycine max*] | 6e-63 |
| Contig 539 | JG293918 | 4 | 1183 | Hsp70-binding protein, putative [*Ricinus communis*] | 7e-100 |
| LT21-260 | JG293919 | 1 | 820 | Hsp70-interacting protein 1 [*Vitis labrusca*] | 1e-57 |
| LT21-1890 | JG293920 | 1 | 662 | Indole-3-acetic acid induced protein ARG-2 homolog [*Glycine max*] | 2e-14 |
| LT21-564 | JG293921 | 1 | 677 | Indole-3-acetic acid induced protein ARG-2 homolog [*Glycine max*] | 6e-20 |
| LT21-1814 | JG293922 | 1 | 1190 | Lectin receptor kinase [*Arabidopsis thaliana*] | 2e-64 |
| Contig 635 | JG293923 | 2 | 488 | Lectin-like protein [*Cicer arietinum*] | 1e-19 |
| LT21-1937 | JG293924 | 1 | 871 | Lectin-like protein [*Cicer arietinum*] | 2e-21 |
| Contig 247 | JG293925 | 2 | 1098 | Leucine-rich repeat family protein [*Glycine max*] | 5e-81 |
| LT21-901 | JG293926 | 1 | 610 | Leucine-rich repeat family protein [*Glycine max*] | 2e-41 |
| Contig 479 | JG293927 | 2 | 617 | Leucine-rich repeat-containing protein, putative [*Ricinus communis*] | 3e-43 |
| Contig 606 | JG293928 | 3 | 1198 | Lipopolysaccharide-binding protein precursor, putative [*Ricinus communis*] | 2e-34 |
| LT21-4637 | JG293929 | 1 | 685 | Lipoxygenase [*Nicotiana attenuata*] | 2e-77 |
| LT21-2966 | JG293930 | 1 | 1049 | Lipoxygenase [*Pisum sativum*] | 9e-110 |
| LT21-513 | JG293931 | 1 | 409 | Lipoxygenase [*Sesbania rostrata*] | 9e-18 |
| LT21-2201 | JG293932 | 1 | 656 | Major allergen Pru ar, putative [*Ricinus communis*] | 3e-45 |
| LT21-4610 | JG293933 | 1 | 608 | Major allergen Pru ar, putative [*Ricinus communis*] | 3e-58 |
| Contig 464 | JG293934 | 2 | 1160 | MATE efflux family protein [*Arabidopsis thaliana*] | 8e-91 |
| LT21-3857 | JG293935 | 1 | 743 | NADH:ubiquinone oxidoreductase family protein [*Arabidopsis thaliana*] | 1e-55 |
| Contig 488 | JG293936 | 2 | 1119 | NADH:ubiquinone oxidoreductase-like [*Solanum tuberosum*] | 3e-92 |
| LT21-2128 | JG293937 | 1 | 1234 | NADP-dependent oxidoreductase, putative [*Arabidopsis thaliana*] | 1e-90 |
| LT21-986 | JG293938 | 1 | 446 | NADPH oxidoreductase homolog [*Cicer arietinum*] | 2e-12 |
| Contig 74 | JG293939 | 10 | 893 | Pathogenesis related protein [*Cicer arietinum*] | 2e-65 |
| Contig 38 | JG293940 | 15 | 1181 | Pathogenesis-related protein [*Pisum sativum*] | 1e-88 |
| Contig 236 | JG293941 | 5 | 845 | Pathogenesis-related protein [*Pisum sativum*] | 9e-83 |
| LT21-1480 | JG293942 | 1 | 1082 | Pathogenesis-related protein [*Pisum sativum*] | 9e-78 |
| LT21-2577 | JG293943 | 1 | 379 | Pathogenesis-related protein [*Pisum sativum*] | 8e-13 |
| Contig 53 | JG293944 | 4 | 608 | Pathogenesis-related protein 4A [*Pisum sativum*] | 3e-73 |
| Contig 70 | JG293945 | 8 | 622 | Pathogenesis-related protein 4A [*Pisum sativum*] | 7e-75 |
| Contig 75 | JG293946 | 6 | 585 | Pathogenesis-related protein 4A [*Pisum sativum*] | 2e-73 |
| Contig 85 | JG293947 | 4 | 570 | Pathogenesis-related protein 4A [*Pisum sativum*] | 1e-74 |
| Contig 465 | JG293948 | 2 | 1218 | Pathogenesis-related protein 4A [*Pisum sativum*] | 1e-48 |
| Contig 564 | JG293949 | 2 | 586 | Pathogenesis-related protein 4A [*Pisum sativum*] | 4e-56 |
| LT21-2718 | JG293950 | 1 | 650 | Pathogenesis-related protein 4A [*Pisum sativum*] | 9e-18 |
| Contig 466 | JG293951 | 2 | 688 | Pathogenesis-related protein PR10 [*Pisum fulvum*] | 1e-73 |
| Contig 642 | JG293952 | 2 | 1218 | Peroxidase [*Phaseolus lunatus*] | 7e-95 |
| LT21-3950 | JG294124 | 1 | 261 | Peroxidase [*Pisum sativum*] | 2e-34 |
| Contig 49 | JG293953 | 6 | 1173 | Peroxidase [*Trifolium repens*] | 3e-108 |
| LT21-724 | JG293954 | 1 | 1128 | Peroxidase 3 precursor [*Phaseolus vulgaris*] | 8e-96 |
| LT21-3446 | JG293955 | 1 | 658 | Peroxiredoxin [*Pisum sativum*] | 1e-82 |
| LT21-3590 | JG293956 | 1 | 633 | Peroxisomal small heat shock protein [*Glycine max*] | 2e-38 |
| LT21-4291 | JG293957 | 1 | 1122 | Peroxisomal small heat shock protein [*Glycine max*] | 5e-30 |
| Contig 611 | JG293958 | 2 | 1151 | Phenylalanine ammonia-lyase 1 [*Manihot esculenta*] | 3e-63 |
| LT21-2027 | JG293959 | 1 | 184 | Phenylalanine ammonia-lyase, putative [*Ricinus communis*] | 1e-112 |
| LT21-1589 | JG293960 | 1 | 1200 | Pheromone receptor-like protein [*Quercus robur*] | 8e-13 |
| LT21-1918 | JG293961 | 1 | 487 | Pheromone receptor-like protein [*Quercus robur*] | 5e-12 |
| Contig 594 | JG293962 | 2 | 1161 | Phosphatidylcholine transfer protein, putative [*Ricinus communis*] | 4e-85 |
| Contig 61 | JG293963 | 2 | 1226 | Phosphosulfolactate synthase-related protein [*Solanum lycopersicum*] | 4e-61 |
| LT21-1987 | JG293964 | 1 | 1160 | Polyubiquitin (ubq10) [*Arabidopsis thaliana*] | 2e-113v |
| LT21-2553 | JG293965 | 1 | 452 | Polyubiquitin [*Capsicum chinense*] | 2e-42 |
| LT21-1364 | JG293966 | 1 | 1192 | Polyubiquitin [*Elaeagnus umbellata*] | 9e-124 |
| LT21-3803 | JG293967 | 1 | 1148 | Polyubiquitin [*Plantago major*] | 4e-88 |
| LT21-4579 | JG293968 | 1 | 412 | Putative defense associated acid phosphatase [*Phaseolus vulgaris*] | 2e-27 |
| LT21-4030 | JG293969 | 1 | 512 | Putative defensin 2.1 precursor [*Medicago sativa*] | 5e-27 |
| LT21-850 | JG293970 | 1 | 1084 | Putative disease resistance protein [*Arabidopsis thaliana*] | 2e-64 |
| LT21-407 | JG293971 | 1 | 1167 | Putative ethylene responsive factor [*Vicia faba*] | 6e-16 |
| LT21-3013 | JG293972 | 1 | 708 | Resistance protein [*Vitis vinifera*] | 4e-34 |
| LT21-2163 | JG293973 | 1 | 1164 | Resistance protein MG13 [*Glycine max*] | 1e-27 |
| LT21-235 | JG293974 | 1 | 616 | Resistance protein MG55 [*Glycine max*] | 1e-34 |
| LT21-2737 | JG293975 | 1 | 578 | S-adenosylmethionine decarboxylase [*Prunus persica*] | 1e-37 |
| Contig 359 | JG293976 | 5 | 1201 | S-adenosylmethionine synthetase 3 [*Populus trichocarpa*] | 2e-132 |
| LT21-2733 | JG293977 | 1 | 459 | S-adenosylmethionine synthetase 5 [*Populus trichocarpa*] | 6e-58 |
| LT21-854 | JG293978 | 1 | 618 | Senescence-associated protein [*Arabidopsis thaliana*] | 1e-32 |
| LT21-2806 | JG293979 | 1 | 820 | Senescence-associated protein DIN1, putative [*Ricinus communis*] | 6e-48 |
| LT21-2987 | JG293980 | 1 | 1149 | Senescence-inducible chloroplast stay-green protein [*Pisum sativum*] | 7e-112 |
| LT21-3691 | JG293981 | 1 | 666 | Senescence-related protein [*Camellia sinensis*] | 3e-18 |
| Contig 440 | JG293982 | 2 | 1033 | Similar to senescence-associated protein [*Arabidopsis thaliana*] | 1e-76 |
| LT21-1725 | JG293983 | 1 | 991 | Similar to senescence-associated protein [*Arabidopsis thaliana*] | 5e-27 |
| Contig 24 | JG293984 | 2 | 716 | Stem-specific protein TSJT1, putative [*Ricinus communis*] | 3e-26 |
| Contig 227 | JG293985 | 4 | 1118 | Stem-specific protein TSJT1, putative [*Ricinus communis*] | 1e-72 |
| Contig 624 | JG293986 | 2 | 914 | Syringolide-induced protein 13-1-1 [*Glycine max*] | 1e-56 |
| LT21-4710 | JG293987 | 1 | 408 | Syringolide-induced protein 13-1-1 [*Glycine max*] | 2e-11 |
| Contig 303 | JG293988 | 2 | 845 | Syringolide-induced protein 14-1-1 [*Glycine max*] | 4e-22 |
| LT21-1439 | JG293989 | 1 | 1165 | Syringolide-induced protein 14-1-1 [*Glycine max*] | 1e-20 |
| LT21-3787 | JG293990 | 1 | 556 | Syringolide-induced protein B13-1-1 [*Glycine max*] | 4e-59 |
| LT21-4659 | JG293991 | 1 | 1114 | Syringolide-induced protein B13-1-1 [*Glycine max*] | 4e-117 |
| Contig 337 | JG293992 | 5 | 923 | Syringolide-induced protein B13-1-9 [*Glycine max*] | 4e-39 |
| LT21-370 | JG293993 | 1 | 640 | Syringolide-induced protein B13-1-9 [*Glycine max*] | 6e-35 |
| Contig 57 | JG293994 | 3 | 880 | Thaumatin-like protein [*Arachis diogoi*] | 4e-69 |
| Contig 31 | JG293995 | 21 | 884 | Thaumatin-like protein [*Pyrus pyrifolia*] | 5e-100 |
| Contig 52 | JG293996 | 28 | 877 | Thaumatin-like protein [*Pyrus pyrifolia*] | 5e-89 |
| Contig 62 | JG293997 | 48 | 866 | Thaumatin-like protein [*Pyrus pyrifolia*] | 2e-96 |
| Contig 252 | JG293998 | 9 | 887 | Thaumatin-like protein [*Pyrus pyrifolia*] | 1e-97 |
| Contig 256 | JG293999 | 2 | 1136 | Thaumatin-like protein [*Pyrus pyrifolia*] | 3e-94 |
| Contig 541 | JG294000 | 2 | 1204 | Thaumatin-like protein [*Pyrus pyrifolia*] | 3e-87 |
| Contig 637 | JG294001 | 2 | 1199 | Thaumatin-like protein [*Pyrus pyrifolia*] | 3e-87 |
| Contig 644 | JG294002 | 2 | 1123 | Thaumatin-like protein [*Pyrus pyrifolia*] | 1e-82 |
| LT21-1257 | JG294003 | 1 | 669 | Thaumatin-like protein [*Pyrus pyrifolia*] | 7e-38 |
| LT21-258 | JG294004 | 1 | 600 | Thaumatin-like protein [*Pyrus pyrifolia*] | 2e-37 |
| LT21-495 | JG294005 | 1 | 1164 | Thaumatin-like protein [*Vitis vinifera*] | 5e-100 |
| Contig 19 | JG294006 | 2 | 1167 | Thaumatin-like protein PR-5a [*Cicer arietinum*] | 9e-69 |
| Contig 366 | JG294007 | 3 | 795 | Thaumatin-like protein PR-5a [*Cicer arietinum*] | 2e-73 |
| Contig 573 | JG294008 | 2 | 765 | Thaumatin-like protein PR-5a [*Cicer arietinum*] | 8e-79 |
| Contig 653 | JG294009 | 2 | 1059 | Thaumatin-like protein PR-5a [*Cicer arietinum*] | 3e-74 |
| LT21-3518 | JG294010 | 1 | 769 | Thaumatin-like protein PR-5a [*Cicer arietinum*] | 3e-76 |
| Contig 323 | JG294011 | 3 | 495 | Type 1 metallothionein [*Pisum sativum*] | 1e-17 |
| Contig 658 | JG294012 | 2 | 377 | Type 1 metallothionein [*Pisum sativum*] | 5e-15 |
| Contig 7 | JG294013 | 4 | 646 | Type 2 metallothionein [*Pisum sativum*] | 3e-18 |
| Contig 557 | JG294014 | 2 | 899 | Ubiquitin conjugating enzyme [*Pisum sativum*] | 6e-77 |
| Contig 347 | JG294015 | 3 | 1167 | Ubiquitin-conjugating enzyme [*Arachis hypogaea*] | 2e-80 |
| LT21-2715 | JG294016 | 1 | 342 | Ubiquitin-conjugating enzyme m, putative [*Ricinus communis*] | 3e-35 |
| LT21-1089 | JG294017 | 1 | 857 | Ubiquitin-protein ligase, putative [*Ricinus communis*] | 6e-38 |
| LT21-2150 | JG294018 | 1 | 1132 | Universal stress protein (USP) family protein [*Arabidopsis thaliana*] | 6e-54 |
| Contig 100 | JG294019 | 2 | 759 | Universal stress protein [*Arachis hypogaea*] | 4e-72 |
| LT21-4014 | JG294020 | 1 | 567 | Universal stress protein 1 [*Gossypium arboreum*] | 5e-44 |
| Contig 361 | JG294021 | 2 | 640 | Universal stress protein-like protein [*Astragalus sinicus*] | 2e-35 |
| LT21-1477 | JG294022 | 1 | 1195 | Drought-induced protein 1 [*Glycine latifolia*] | 5e-29 |
| Contig 232 | JG294023 | 2 | 702 | Water stress-induced protein, putative [*Arabidopsis thaliana*] | 6e-06 |
| Contig 39 | JG294024 | 4 | 482 | Stress associated endoplasmic reticulum protein, putative [*Ricinus communis*] | 5e-15 |
| Contig 80 | JG294025 | 2 | 1049 | Stress induced protein [*Vitis vinifera*] | 9e-22 |
| LT21-928 | JG294026 | 1 | 1148 | Chitin-inducible gibberellin-responsive protein, putative [*Ricinus communis*] | 7e-33 |
| LT21-1855 | JG294027 | 1 | 1143 | Auxin-induced protein 5NG4, putative [*Ricinus communis*] | 7e-53 |
| Contig 461 | JG294028 | 2 | 1085 | Hypoxia induced protein conserved region containing protein [*Zea mays*] | 1e-10 |
| Contig379 | JG294029 | 3 | 1161 | Altered response to gravity (arg1), plant, putative [*Ricinus communis*] | 3e-96 |
| LT21-1827 | JG294030 | 1 | 1074 | Hairpin-inducing protein [*Casuarina glauca*] | 7e-18 |
| LT21-573 | JG294031 | 1 | 744 | Hypersensitive-induced response protein [*Carica papaya*] | 2e-60 |
| LT21-2154 | JG294032 | 1 | 1200 | Putative Pi starvation-induced protein [*Cicer arietinum*] | 4e-28 |
| Contig 462 | JG294033 | 2 | 922 | Wound-responsive protein-related [*Arabidopsis thaliana*] | 1e-10 |
| LT21-1141 | JG294034 | 1 | 666 | Wound-responsive family protein [*Arabidopsis lyrata* ssp. *lyrata*] | 4e-15 |
| Contig 218 | JG294035 | 2 | 705 | WRKY transcription factor, putative [*Ricinus communis*] | 6e-47 |
| LT21-3030 | JG294036 | 1 | 1204 | WRKY transcription factor, putative [*Ricinus communis*] | 4e-39 |
| LT21-2845 | JG294037 | 1 | 958 | Zinc finger protein-related [*Arabidopsis thaliana*] | 1e-44 |
| Contig 105 | JG294038 | 2 | 1061 | 1-aminocyclopropanecarboxylic acid oxidase [*Medicago truncatula*] | 8e-72 |
| Contig 81 | JG294039 | 3 | 1174 | Properoxidase [*Picea abies*] | 3e-57 |
| LT21-1588 | JG294040 | 1 | 423 | Tumor-related protein [*Nicotiana tabacum*] | 3e-26 |
| LT21-1651 | JG294041 | 1 | 1183 | Putative peroxidase [*Solanum tuberosum*] | 4e-12 |
| LT21-236 | JG294042 | 1 | 338 | Alternative oxidase [*Crocus sativus*] | 6.8 |
| LT21-2723 | JG294043 | 1 | 656 | Quinone oxidoreductase, putative [*Ricinus communis*] | 2e-55 |
| Contig 237 | JG294044 | 2 | 1110 | Putative quinone oxidoreductase [*Cicer arietinum*] | 2e-85 |
| LT21-378 | JG294045 | 1 | 1164 | Cationic peroxidase 2 precursor, putative [*Ricinus communis*] | 3e-110 |
| LT21-4112 | JG294046 | 1 | 870 | Allene-oxide cyclase [*Medicago truncatula*] | 7e-70 |
| Contig 185 | JG294047 | 5 | 1133 | Cytosolic ascorbate peroxidase [*Vigna unguiculata*] | 8e-119 |
| LT21-4420 | JG294048 | 1 | 280 | 2-nitropropane dioxygenase precursor, putative [*Ricinus communis*] | 5e-20 |
| LT21-1402 | JG294049 | 1 | 1141 | GTP binding protein gamma subunit [*Brassica napus*] | 3e-04 |
| Contig 356 | JG294050 | 2 | 1176 | Matrix metalloprotease 1 [*Nicotiana benthamiana*] | 8e-22 |
| Contig 129 | JG294051 | 2 | 1192 | Protease inhibitor/seed storage/lipid transfer protein family protein [*Tamarix hispida*] | 6e-12 |
| Contig 36 | JG294052 | 4 | 603 | Putative protease inhibitor [*Glycine max*] | 2e-19 |
| LT21-2902 | JG294053 | 1 | 1189 | Thiolprotease [*Pisum sativum*] | 5e-138 |
| Contig 663 | JG294054 | 2 | 1036 | Cinnamoyl-coa reductase, putative [*Ricinus communis*] | 1e-63 |
| LT21-2986 | JG294055 | 1 | 720 | Cinnamyl alcohol dehydrogenase [*Leucaena leucocephala*] | 3e-66 |
| LT21-2646 | JG294056 | 1 | 738 | 14-3-3-like protein [*Cicer arietinum*] | 1e-105 |
| LT21-3798 | JG294057 | 1 | 517 | 2-Cys peroxiredoxin [*Pisum sativum*] | 2e-41 |
| LT21-1721 | JG294058 | 1 | 1027 | 3-hydroxy-3-methylglutaryl coenzyme A synthase [*Salvia miltiorrhiza*] | 2e-110 |
| LT21-1811 | JG294059 | 1 | 1041 | AFR (ATTENUATED FAR-RED RESPONSE) [*Arabidopsis thaliana*] | 1e-21 |
| Contig 598 | JG294060 | 2 | 1216 | AKIN gamma [*Medicago truncatula*] | 3e-74 |
| Contig 324 | JG294061 | 2 | 806 | Alanine aminotransferase 2 [*Glycine max*] | 3e-103 |
| LT21-863 | JG294062 | 1 | 1210 | Alpha-glucan water dikinase, chloroplast precursor, putative [*Ricinus communis*] | 1e-112 |
| LT21-4935 | JG294063 | 1 | 647 | Anamorsin, putative [*Ricinus communis*] | 6e-41 |
| Contig 319 | JG294064 | 3 | 1153 | F-box protein At1g61340 [*Medicago truncatula*] | 1e-20 |
| LT21-4481 | JG294065 | 1 | 956 | Beta-expansin 1a precursor, putative [*Ricinus communis*] | 4e-96 |
| LT21-2991 | JG294066 | 1 | 778 | Beta-glucosidase G3 [*Medicago truncatula*] | 7e-73 |
| Contig 44 | JG294067 | 3 | 846 | Brassinosteroid LRR receptor kinase precursor, putative [*Ricinus communis*] | 3e-106 |
| LT21-999 | JG294068 | 1 | 712 | Calmodulin-related protein, putative [*Arabidopsis thaliana*] | 3e-17 |
| LT21-2545 | JG294069 | 1 | 460 | Calreticulin-1 [*Glycine max*] | 8e-73 |
| Contig 204 | JG294070 | 2 | 735 | Catalase 1 [*Pisum sativum*] | 4e-105 |
| Contig 552 | JG294071 | 2 | 445 | Chain a, high resolution structure of a cherry allergen Pru av 2 | 2e-24 |
| LT21-3851 | JG294072 | 1 | 1156 | Copper chaperone precursor [*Solanum tuberosum*] | 3e-78 |
| LT21-3968 | JG294073 | 1 | 1199 | Desacetoxyvindoline 4-hydroxylase, putative [*Ricinus communis*] | 2e-68 |
| LT21-3910 | JG294074 | 1 | 705 | EDR1 [*Glycine max*] | 1e-65 |
| Contig 528 | JG294075 | 3 | 1182 | Formate dehydrogenase [*Lotus japonicus*] | 3e-119 |
| LT21-2509 | JG294076 | 1 | 341 | Galactose-binding like [*Medicago truncatula*] | 9e-46 |
| LT21-1491 | JG294077 | 1 | 1145 | IFRD protein family [*Arabidopsis thaliana*] | 9e-55 |
| Contig 662 | JG294078 | 2 | 851 | Kunitz trypsin inhibitor 4 [*Arachis hypogaea*] | 5e-60 |
| LT21-1830 | JG294079 | 1 | 424 | LITAF-domain-containing protein [*Pisum sativum*] | 3e-07 |
| Contig 360 | JG294080 | 2 | 819 | MEE14 (maternal effect embryo arrest 14) [*Arabidopsis thaliana*] | 1e-48 |
| LT21-1693 | JG294081 | 1 | 1165 | Metallopeptidase family M24 containing protein, expressed [*Oryza sativa*] | 4e-82 |
| LT21-987 | JG294082 | 1 | 1140 | MLO1 [*Pisum sativum*] | 2e-129 |
| LT21-173 | JG294083 | 1 | 894 | Molecular chaperone Hsp90-2 [*Nicotiana benthamiana*] | 4e-94 |
| Contig 153 | JG294084 | 3 | 1187 | Monodehydroascorbate reductase I [*Pisum sativum*] | 3e-110 |
| LT21-3068 | JG294085 | 1 | 946 | MTD1 [*Medicago truncatula*] | 3e-17 |
| LT21-4393 | JG294086 | 1 | 660 | NOI protein [*Arabidopsis thaliana*] | 3e-26 |
| LT21-2831 | JG294087 | 1 | 880 | N-rich protein [*Glycine max*] | 1e-29 |
| LT21-2701 | JG294088 | 1 | 362 | NSL1 (necrotic spotted lesions 1) [*Arabidopsis thaliana*] | 7e-33 |
| LT21-2340 | JG294089 | 1 | 842 | Nteig-E80 [*Nicotiana tabacum*] | 5e-46 |
| Contig 178 | JG294090 | 2 | 1141 | PAR-1c [*Nicotiana tabacum*] | 2e-53 |
| Contig 429 | JG294091 | 2 | 1032 | Peptidase C1A, papain; Somatotropin hormone; Peptidase C1, propeptide | 2e-108 |
| Contig 104 | JG294092 | 4 | 1168 | Plastid jasmonates ZIM-domain protein [*Hevea brasiliensis*] | 3e-46 |
| LT21-4521 | JG294125 | 1 | 1076 | Polygalacturonase inhibiting protein [*Pisum sativum*] | 3e-113 |
| Contig 15 | JG294093 | 2 | 1169 | Potyviral capsid protein interacting protein 1 [*Nicotiana tabacum*] | 6e-08 |
| Contig 171 | JG294094 | 2 | 750 | PR10-1 protein [*Medicago truncatula*] | 8e-47 |
| Contig 137 | JG294095 | 7 | 930 | PR-5 protein [*Glycine max*] | 5e-85 |
| LT21-2439 | JG294096 | 1 | 1039 | Predicted protein [*Populus trichocarpa*] | 4e-81 |
| LT21-1235 | JG294097 | 1 | 517 | Pshsp71.2 [*Pisum sativum*] | 1e-56 |
| Contig 545 | JG294098 | 4 | 1153 | Putative chalcone synthase [*Medicago truncatula*] | 3e-162 |
| Contig 407 | JG294099 | 2 | 549 | Putative galactinol synthase [*Pisum sativum*] | 3e-33 |
| Contig 87 | JG294100 | 2 | 1154 | 22.7 kda class IV heat shock protein | 1e-36 |
| Contig 164 | JG294101 | 2 | 458 | Metallothionein-like protein 1A [*Vicia faba*] | 1e-08 |
| LT21-304 | JG294102 | 1 | 546 | Ribosomal protein l27a-like protein [*Solanum tuberosum*] | 1e-60 |
| LT21-1495 | JG294103 | 1 | 605 | Salicylic acid methyl transferase-like protein [*Glycine max*] | 1e-11 |
| LT21-4586 | JG294104 | 1 | 1185 | 6a-hydroxymaackiain methyltransferase [*Pisum sativum*] | 2e-96 |
| Contig 547 | JG294105 | 2 | 1172 | Sensor histidine kinase, putative [*Ricinus communis*] | 2e-86 |
| Contig 310 | JG294106 | 2 | 1185 | SKP1-like b [*Medicago truncatula*] | 5e-56 |
| LT21-3520 | JG294107 | 1 | 522 | Snakin-2 precursor [*Solanum lycopersicum*] | 2e-21 |
| LT21-1795 | JG294108 | 1 | 1188 | Speckle-type POZ protein, putative [*Ricinus communis*] | 7e-53 |
| Contig 179 | JG294109 | 9 | 1254 | STS14 protein precursor, putative [*Ricinus communis*] | 2e-42 |
| Contig 459 | JG294110 | 5 | 1147 | Syntaxin, plant, putative [*Ricinus communis*] | 1e-40 |
| LT21-1024 | JG294111 | 1 | 1163 | Terpene synthase [*Medicago truncatula*] | 2e-113 |
| LT21-820 | JG294112 | 1 | 1097 | TIR [*Medicago truncatula*] | 6e-44 |
| Contig 186 | JG294113 | 2 | 1140 | Tir-nbs-lrr resistance protein [*Populus trichocarpa*] | 8e-28 |
| LT21-4470 | JG294114 | 1 | 627 | Toll interleukin receptor [*Glycine max*] | 1e-43 |
| LT21-1824 | JG294115 | 1 | 769 | Type IIB calcium atpase [*Medicago truncatula*] | 3e-04 |
| LT21-3464 | JG294116 | 1 | 1172 | UDP-glucosyltransferase, putative [*Ricinus communis*] | 3e-76 |
| LT21-1803 | JG294117 | 1 | 390 | Ultraviolet-B-inducible glucanase [*Pisum sativum*] | 5e-24 |
| LT21-2579 | JG294118 | 1 | 381 | UP-9A [*Nicotiana tabacum*] | 6e-10 |
| Contig 144 | JG294119 | 5 | 1212 | Chalcone reductase [*Medicago sativa*] | 3e-148 |
| Contig 169 | JG294120 | 5 | 1079 | 6a-hydroxymaackiain methyltransferase [*Pisum sativum*] | 4e-148 |
| Contig 425 | JG294121 | 2 | 1209 | Chalcone reductase [*Pueraria montana* var. Lobata] | 9e-113 |
| Contig 566 | JG294122 | 2 | 1150 | Chalcone isomerase A [*Glycine max*] | 7e-104 |
| LT21-1380 | JG294123 | 1 | 1124 | Chalcone synthase [*Lupinus luteus*] | 2e-62 |
| LT21-1683 | JG293479 | 1 | 1197 | Endoplasmic reticulum HSC70-cognate binding protein precursor [*Glycine max*] | 5e-18 |
