# Supplementary material for: Identification of Lens culinaris defense genes responsive to the anthracnose pathogen Colletotrichum truncatum
Source: BMC Genet. 2013 Apr 30;14:31. doi: 10.1186/1471-2156-14-31 (PMC3666911; doi:10.1186/1471-2156-14-31)
Supplement: Additional file 2 — List of primers. Oligonucleotide sequence of reference and target genes used in qRT-PCR. [file 1471-2156-14-31-S2.docx]

| \| Primer name \| Oligonucleotide sequence (5’→3’) \| \| --- \| --- \| \| EF1-αF \| TGTCGACTCTGGGAAGTCAA \| \| EF1-αR \| CTCTTTCCCTTTCAGCCTTG \| \| LT21-1990F \| ATTTGGCGACTTTGAAGGAC \| \| LT21-1990R \| ATGTCATTGGGAAGCCTTGT \| \| Contig 186F \| TTCAAAGGCAGTGATATCCG \| \| Contig 186R \| TCCTCCAAACACCAATACGA \| |  |
| --- | --- | --- | --- | --- | --- | --- | --- | --- | --- | --- | --- | --- | --- | --- | --- |
|  |  |
